# Synthesis of carbon-based nanomaterials and their application in pollution management

**DOI:** 10.1039/d1na00843a

**Published:** 2022-01-20

**Authors:** Zhixin Liu, Qian Ling, Yawen Cai, Linfeng Xu, Jiahao Su, Kuai Yu, Xinyi Wu, Jiayi Xu, Baowei Hu, Xiangke Wang

**Affiliations:** School of Life Science, Shaoxing University Huancheng West Road 508 Shaoxing 312000 China hbw@usx.edu.cn xkwang@ncepu.edu.cn

## Abstract

With the fast development of industry, large amounts of organic and inorganic pollutants are inevitably released into the natural environment, which results in the pollution of the environment and are thereby dangerous to human health. The efficient elimination of these pollutants is crucial to environment protection and human health. The high sorption capacity of carbon-based materials and high photocatalytic ability of carbon-based composites result in the application of carbon-based materials in environmental pollution cleanup. In this review article, we summarized recent studies on the synthesis of carbon-based materials, and their application in the sorption of organic and inorganic pollutants, the photocatalytic degradation of organic pollutants, and the *in situ* photocatalytic reduction–solidification of heavy metal ions. The sorption method is useful to remove pollutants from aqueous solutions. The sorption-photocatalytic degradation of organic pollutants is applicable, especially at low concentrations, whereas the catalytic reduction of metal ions is the best method for the *in situ* immobilization of high valent metal ions under complicated conditions. The interaction mechanism is discussed using advanced spectroscopy analysis and theoretical calculations, and at the end the challenges in the future are described.

## Abstract 2

With the fast development of industry, large amounts of organic and inorganic pollutants are inevitably released into the natural environment, which results in the pollution of the environment and are thereby dangerous to human health.

## Graphical abstract

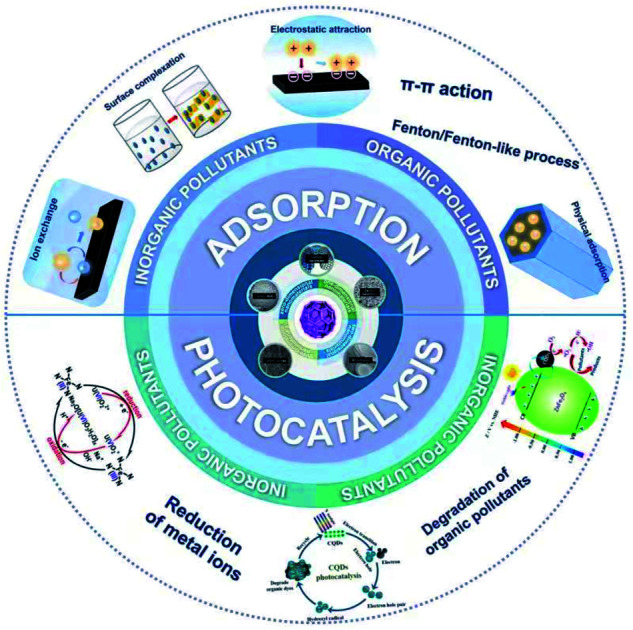

## 1. Introduction

The development of modern society, the acceleration of urbanization, the growth of industrial production and the modernization of transportation have brought serious environmental pollution issues. The disturbance of the natural environment caused by human productivity and daily living activities is increasing. Harmful pollutants such as antibiotics, pesticides, dyes, heavy metals, greenhouse gases, endocrine-disrupting chemicals and volatile organic compounds are discharged into water, soil and air, posing a serious threat to the environment and human health.^1,2^ According to the “2018 UN World Water Development Report”, about 80% of wastewater from industrial and municipal activities are released into nature without pretreatment, directly or indirectly causing the deterioration of water quality.^3^ Most wastewater contains toxic levels of metal ions, organic molecules, dyes and other carcinogenic chemicals, and should be pretreated before releasing into the natural environment.^4^ Scientists have made great efforts to find many effective materials to remove pollutants from wastewater, such as clay minerals, carbon materials, and natural/man-made nanomaterials.^5,6^ Different kinds of these materials have been reviewed in the elimination of pollutants, and showed advantages/disadvantages in real applications.^7–10^

**Fig. 1:**
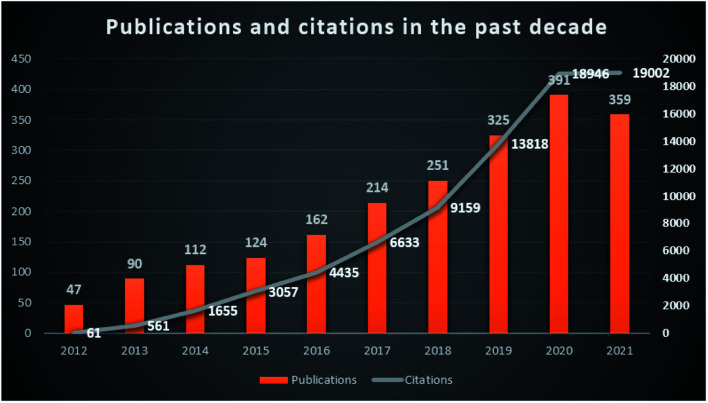
Publications and citations of carbon-based nanomaterials in the environmental field in the past decade from the Web of Science.

Carbon-based nanomaterials have large specific surface areas, excellent acid stability, and thermal resistance, which have drawn much attention in the field of pollution treatment because of their excellent physicochemical properties.^11^ It is found that carbon materials can effectively remove pollutants such as nitric oxide, hydrogen sulfide, heavy metals, dyes and drug compounds from the environment, with adsorption efficiency > 80% or photocatalytic degradation efficiency > 98%.^1^ There has been a steady increase in reports of carbon-based nanomaterials in pollutant treatment over the last decade (2012–2021) (Fig. 1, retrieved 2012–2021 publications from the Web of Science Core Collection database using keywords of carbon based or carbon-based, nanomaterials or nanomaterial, and pollutants or environment or environmental). It turned out that more than 2000 records matched the selected keywords. Comparing the annual publications and citations from 2012 to 2021, it shows the research interest increase of carbon-based nanomaterials in the environmental pollution treatment field.

**Fig. 2:**
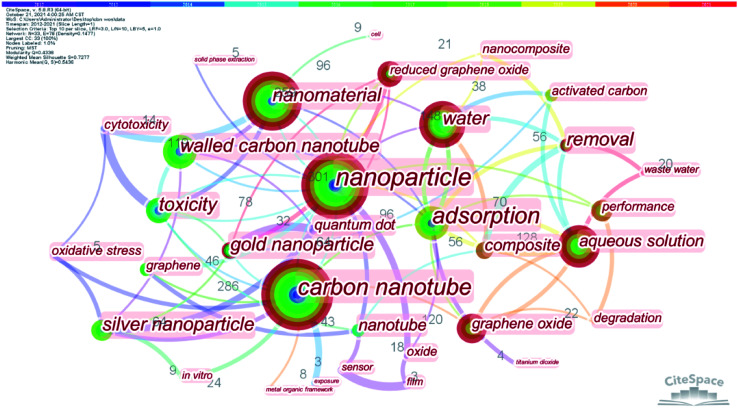
The analysis of keywords co-found.

**Fig. 3:**
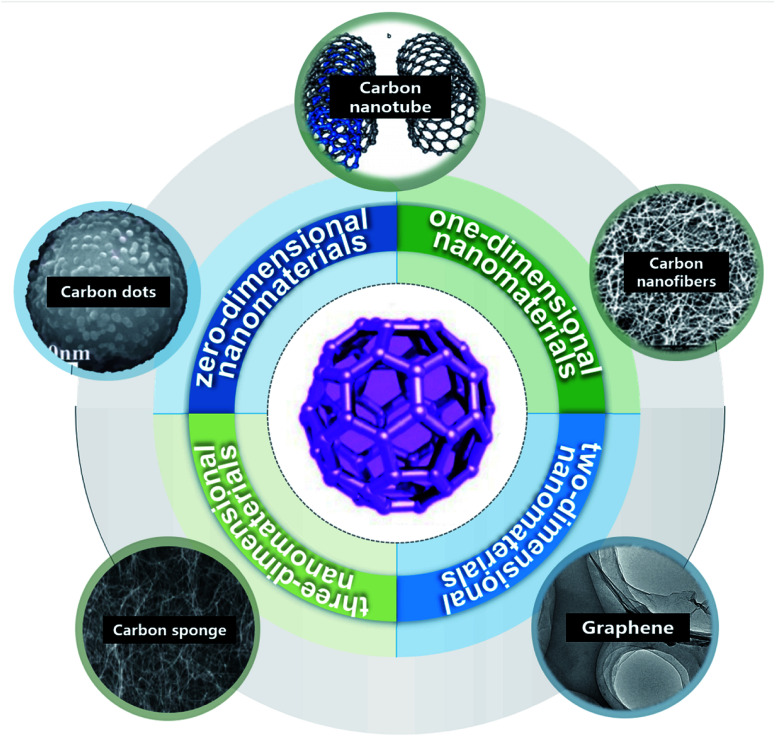
Types of carbon-based nanomaterials: CDs “reproduced from ref. 15 with permission from Elsevier, copyright 2018”, CNTs “reproduced from ref. 11 with permission from Chinese Academy of Science, copyright 2017”, CNFs “reproduced from ref. 16 with permission from Frontier, copyright 2019”, graphene “reproduced from ref. 18 with permission from American Chemical Society, copyright 2011”, carbon sponges “reproduced from ref. 20 with permission from Wiley, copyright 2010” and Carbon-60 “reproduced from ref. 12 with permission from World Scientific, copyright 2021”.

From the bibliometric analysis of keywords co-found by Cite Space it is seen that several keywords (such as nanoparticle, carbon nanotubes (CNTs), nanomaterial, water, aqueous solution, graphene oxides (GOs) and multi-walled CNTs (MWCNTs)) were frequently used in the published papers, indicating that these carbon nanomaterials have received more attention in current research on carbon-based nanomaterials (Fig. 2). According to their shape, size and dimension, carbon nanomaterials^12,13^ can be divided into zero-dimensional nanomaterials (buckminsterfullerene^14^ and carbon dots (CDs)^15^), one-dimensional nanomaterials (CNTs^11^ and carbon nanofibers (CNFs)^16^), two-dimensional nanomaterials (graphene^17,18^) and three-dimensional nanomaterials such as carbon sponges^1,19,20^ (Fig. 3).

As a kind of zero-dimensional nanomaterial, C_60_ has gained much attention owing to its unique structure and performance since its discovery in 1985. After several years of in-depth research, C_60_ was found to have great potential as a cornerstone of molecular engineering, new material synthesis, supramolecular chemistry and pharmaceutical chemistry.^21^ CDs and their composites are easy to obtain raw materials, and convenient to synthesize and function, and have unique optical properties and rich functional groups, which make them widely applied in electronic equipment, biomedicine, environmental pollution treatment and many other fields.^22^ One-dimensional carbon nanomaterials mainly consist of CNTs and CNFs. CNTs are tubular structures formed from round graphene sheets and can be divided into single-walled CNTs, double-walled CNTs or MWCNTs. MWCNTs are more easy to synthesize and can effectively remove metal ions such as Cr(iii).^23^ CNTs have high mechanical strength and chemical stability and have broad application prospects. As a typical two-dimensional nanomaterial, graphene is composed of a single layer or several layers of graphite, and has broad application prospects in environmental remediation. GOs are widely used for the treatment of wastewater, heavy metal ions, and organic compounds.^24^ CNT sponge is a classic three-dimensional nanomaterial, which is a kind of high porous material with many different properties such as flexible deformation and shape recovery. CNT sponges provide a common platform for the design and manufacture of functional composites at various levels by introducing polymer or inorganic guests.^18^

In this review article, we mainly summarized the recent studies on the synthesis of carbon-based nanomaterials, the adsorption mechanism of organic pollutants and heavy metal ions, and the photocatalytic degradation of organic pollutants and *in situ* (photo)catalytic reduction–solidification of high valent heavy metal ions to low valent metal ions. The interaction mechanism was discussed from advanced spectroscopy analysis and theoretical calculations in detail.

## 2. Synthesis of carbon-based nanomaterials

### 2.1 Synthesis of low dimensional carbon nanomaterials

#### 2.1.1 Carbon dots

**Fig. 4:**
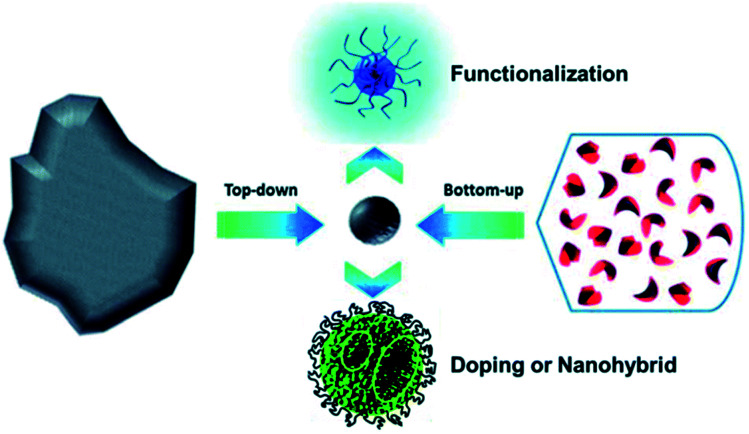
Schematic illustration of CD preparation *via* “top-down” and “bottom-up” approaches, and modification including functionalization, doping and nanohybrids. “Reproduced from ref. 25 with permission from Royal Society of Chemistry, copyright 2014”.

**Fig. 5:**
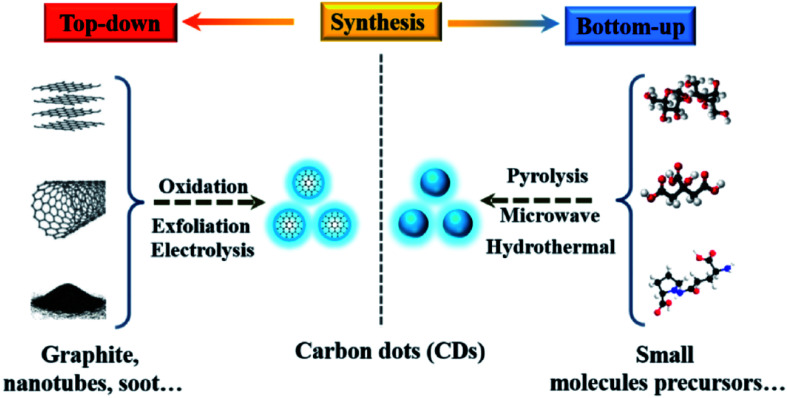
Different synthesis methods of CDs. “Reproduced from ref. 26 with permission from Elsevier, copyright 2021”.

The synthesis methods of CDs include hydrothermal/solvothermal, microwave, electrochemical, microplasma and chemical oxidation methods. The properties of CDs synthesized by different methods are different.^22^ These methods can be generally divided into “top-down” and “bottom-up” approaches, *i.e.*, one is to use physical or chemical methods to cut a large number of carbonaceous materials into CDs, including electrochemical synthesis, chemical oxidation and solvent heat treatment; the other is to synthesize CDs from a precursor, using the method of hydrothermal treatment, microwave assisted synthesis, thermal decomposition or carbonization (Fig. 4 and 5).^25,26^ According to the structures, properties and preparation methods, CDs are mainly divided into carbon quantum dots (CQDs), graphene quantum dots (GQDs) and carbonized polymer dots (CPDs).^27^

**Fig. 6:**
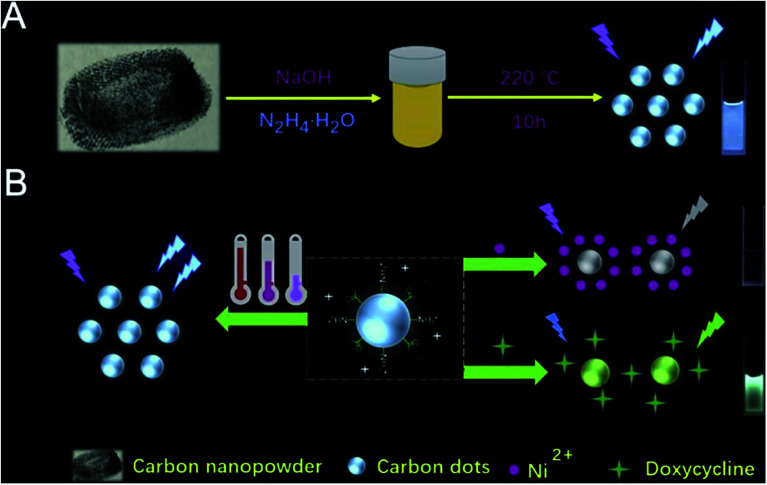
(A) Schematic illustration of synthesizing CDs; (B) description of sensing detection of Ni(ii) and doxycycline “reproduced from ref. 30 with permission from Elsevier, copyright 2019”.

Specific CDs were prepared by optimizing the synthesis conditions such as time, temperature and voltage. To obtain independent purified and monodisperse CDs, the resulting products are usually further purified using either simple treatment steps (*e.g.* centrifugation, dialysis and filtration) or complex and precise treatment processes (*e.g.* electrophoresis, silica gel column chromatography, high performance liquid chromatography, *etc.*).^28^ Hydrothermal/solvothermal synthesis is the most commonly used method for CD synthesis. The usual processes are as follows: the raw material is dissolved in water or solvent, poured into a Teflon-lined stainless autoclave, placed in an oven and heated at a certain temperature for a period of time to obtain CDs. This method has many advantages, such as a simple process and easy operation, uniform synthesis of CDs with high yield, and easy to add other elements and adjust the diversity of CDs.^29^ Su *et al.*^30^ synthesized multifunctional CDs using one-step hydrothermal synthesis of nano-toners. By fluorescence changes, the proposed CDs exhibited multiple functions of sensing temperature (induced fluorescence enhancement, achieving a wide linear range from 25 °C to 95 °C), Ni(ii) (specific fluorescence quenching, detection range of 80–6000 μM) and doxycycline (fluorescence from blue to green, 10 to 1000 μM can be visually observed) by virtue of three different routes (Fig. 6).^30^ The preparation of CQDs by electrochemical carbonization was rarely reported. Zhang and co-workers^31^ prepared CQDs by electrochemically carbonizing alcohols of low molecular weight, with two platinum plates as working and auxiliary electrodes and a calomel electrode mounted on a freely adjustable Luggin capillary as a reference electrode. Using electrochemical carbonization, alcohols were converted to CQDs. The size and graphitization of CQDs increased with the increase of application potential.^25^ The CDs showed high sorption capacity of pollutants because of their high specific surface area, stability and abundant functional groups, and therefore attract much attention in environmental pollution management.

#### 2.1.2 Carbon fibers & CNTs

**Fig. 7:**
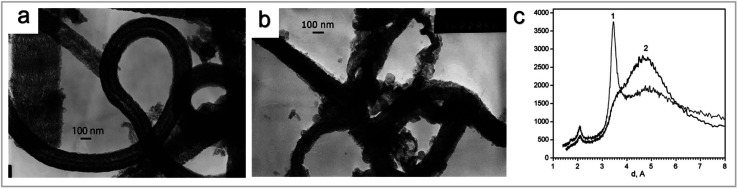
(a) Typical electron image of CNF obtained by a CVD method from acetylene on the Fe/C/SiO_2_ catalyst. (b) Typical electron image of CNF activated melted KOH modified with 2 wt% of copper. (c) XRD patterns of CNF (curve 1) and CNF activated with melted KOH (curve 2) “reproduced from ref. 34 with permission from Elsevier, copyright 2008”.

In the 1960s, carbon fibers (CNFs) became one of the most important industrial materials of modern science and technology. CNFs were produced by melt spinning processes from carbon precursors. Polyacrylonitrile (PAN) was used as the main precursor for various modifications, such as adding additives, oxidation stabilization at low temperature, and stretching during stabilization and carbonization. A catalytic chemical vapor deposition (CVD) process was applied for vapor grown carbon fibers (VGCFs). Thin tubes consisting of straight carbon layers had been found in the center of VGCFs, which were named CNTs. It was reported to be formed *via* arc-discharging. CNTs are considered as important materials for the development of modern nanotechnology in the 21st century.^32^ There are two main methods to synthesized CNFs, *i.e.*, (1) catalytic decomposition of carbon precursors; and (2) carbonization and electrospinning of polymers. The most suitable method is selected according to the desired synthetic CNF properties.^33^ CNFs are synthesized by CVD through decomposition of carbon containing gas catalyzed by metal particles. Common gases are acetylene, ethylene, methane, and propylene. Activated by dissolved KOH, the surface area of the CNFs increased 4–7 times (*i.e.*, from 300–400 to 1700 m^2^ g^−1^) (Fig. 7).^34^ The most commonly used metal catalysts for CNF growth are Fe, Co and Ni. Cr, V and Mo have also been studied.^35,36^ Compared to the production of other similar carbon nanostructures such as single-walled CNTs, the cost effectiveness of mass production of CNFs is significantly improved. This is a particular advantage.^33^ Electrospinning is an effective technique for the production of polymer nanofibers. It is used to produce nanofibers in different forms from tens of nanometers to several microns. A strong electrostatic field is applied to a capillary, which is connected by a polymer solution reservoir.^33^ In electrospinning, polymer fiber morphologies are significantly affected by the polymer solution concentration, working distance, spinning voltage and feeding speed. The fiber size decreases with the increase of carbonization temperature.^37^

Arc discharge, laser evaporation and CVD are three main methods for preparation of single-walled CNTs (SWCNTs).^38^ Arc discharge involves a closed reaction chamber, which is filled with H_2_, He and other gases. A thick graphite rod is used as the cathode, which is filled with Fe, Co, Ni and other metal catalysts as the anode, using the glow between the graphite electrodes to generate electricity. High purity SWCNTs can be synthesized in the inner wall of the reaction vessel. CNTs prepared by the arc discharge method have high yield, high crystallinity and a short preparation time. The layer number of CNTs can be adjusted by the selection of catalyst. The laser evaporation method is similar to arc discharge. The transition metal catalyst and graphite composite rod are vaporized by a laser in a high temperature quartz tube, and the product is deposited on a water-cooled copper column by flowing inert gas to obtain CNTs. This method is not suitable for large-scale production because of its high energy consumption, high preparation cost and difficult popularization. CVD is used to catalyze the pyrolysis of ethanol, ethylene, ethane and other hydrogen compounds to produce CNTs at a high temperature.^11^ CVD can produce CNTs in a large scale, including fixed bed CVD, fluidized bed CVD, plasma enhanced CVD and aerosol-assisted CVD.^39^

### 2.2 Synthesis of high dimensional carbon nanomaterials

Graphene nanosheets are made up of a single-atom-thick hexagonal lattice of carbon atoms, each covalently bound to three others, whereas graphite consists of many graphene nanosheets with van der Waals bonding. In 2004, Geim and Novoselov^40^ prepared graphene using Scotch tape and graphite, and thereby they were awarded the Nobel Prize for their work. Since the discovery, research on graphene had proliferated. Graphene has high strength and superconductivity.^41,42^ It has many advantages for water treatment because of its thinness, light weight, photoactivity, high surface area and chemical stability. With these advantages, graphene has attracted multidisciplinary interest for many areas.^17^

**Fig. 8:**
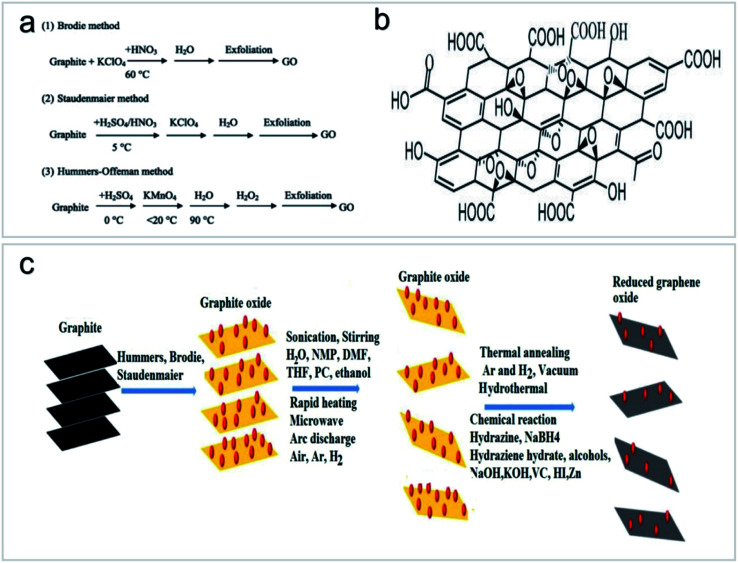
(a) Synthesis processes of GOs “reproduced from ref. 47 with permission from Elsevier, copyright 2013”, (b) structural model of GOs “reproduced from ref. 46 with permission from Elsevier, copyright 2017”, (c) routes to the synthesis of GOs and reduced GOs “reproduced from ref. 46 with permission from Elsevier, copyright 2017”.

Graphene functionalization for environmental applications is mainly performed by chemical methods, including pure chemical processes (*i.e.*, chemical oxidation and deposition) and extended chemical processes (*i.e.*, electrochemistry, sol–gel, microemulsion and hydrothermal methods). These methods can introduce functional groups to carbon nanomaterials to achieve a variety of functions.^43^ GOs have received great attention in the field of environmental science because they can be prepared by simple chemical methods and have strong hydrophilic ability to form a stable GO sol suspension. In 1958, Hummers *et al.*^44^ prepared high-quality GOs by treating graphite with a mixture of KMnO_4_, NaNO_3_ and concentrated H_2_SO_4_ (Fig. 8a and b). The application of Hummers method for the preparation of GOs has the advantages of simple reaction, a relatively short time and high safety. After decades of development and improvement, the Hummers method has become a relatively mature method for GO preparation. On this basis, several methods for GO preparation by stripping graphite have been derived, as shown in Fig. 8c.^45,46^

**Fig. 9:**
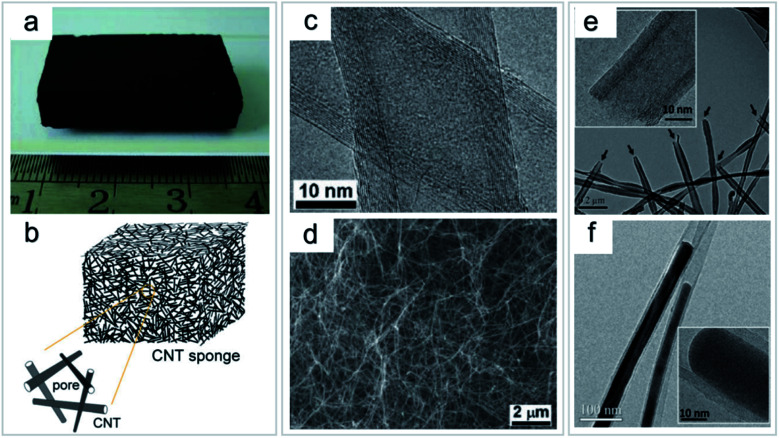
Direct growth of CNT sponge by a CVD process. (a) Photo of an as-grown bulk CNT sponge. (b) Schematic structure of a 3D CNT sponge. (c) TEM image of the multi-walled CNTs. (d) SEM image of the inner part of the sponge showing randomly stacked CNTs. (e) TEM image of CNTs with uniform open-ended structures (arrows); inset shows the HRTEM image. (f) TEM image showing an Fe catalyst encapsulated inside CNTs. Inset shows the high-resolution TEM image. “Reproduced from ref. 20 with permission from Wiley, copyright 2010”.

There are a variety of methods for synthesizing 3D carbon nanostructures. CNT sponge is a high-performance porous material with a variety of different properties such as flexible deformation and shape recovery. It provides a common platform for the design and manufacture of functional composites at all levels by introducing polymers or inorganic guests.^19^ Gui *et al.*^48^ synthesized CNTs by CVD and controlled the synthesis of sponge CNT blocks with isotropy and a flexible independent structure. CNT sponge consists of overlapping CNTs which interweave themselves into a highly porous interconnected 3D framework (Fig. 9).^20,49^

From the abovementioned methods for the synthesis of carbon-based nanomaterials, one can see that it is still difficult to synthesize carbon-based nanomaterials with high quality on a large scale and at a low price. However, for the application of carbon-based nanomaterials in the efficient elimination of pollutants from wastewater, the nanomaterials should have a high specific surface area with large amounts of functional groups and abundant binding sites. The quality of the nanomaterial is not essential for the materials to have high sorption capacity.

## 3. Adsorption of pollutants

The functionalized derivatives of carbon-based nanomaterials have high specific surface areas and abundant adsorption sites, which can form strong complexes with heavy metals and organic molecules on solid surfaces and thereby can remove pollutants efficiently from wastewater.^22,50,51^

### 3.1 Adsorption of metal ions

**Fig. 10:**
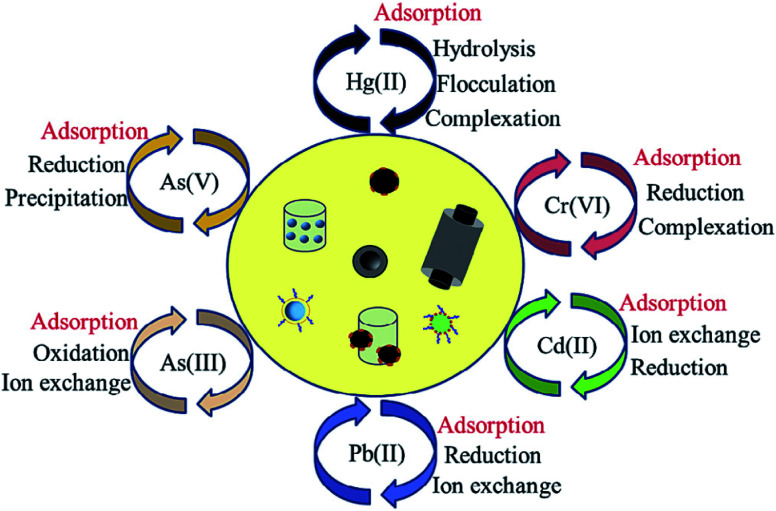
Pathways for aqueous heavy metal ion removal by functionalized nanomaterials “reproduced from ref. 43 with permission from Elsevier, copyright 2018”.

In terms of the adsorption of metal ions from aqueous solutions, physical adsorption, precipitation, sorption/reduction, ion exchange and electrostatic interactions dominate the interaction between heavy metal ions and surface functional groups, which contribute mainly to the uptake of metal ions by carbon nanomaterials, depending on the surface properties of the nanomaterials and functional groups (Fig. 10).^43,52^

In general, the interaction between metal ions and surface oxygen-containing groups plays an important role in the adsorption capacity of carbon-based nanomaterials for heavy metals. CNT raw materials typically require acid treatment or oxidation treatment to increase their surface oxygen-containing functional groups, whereas GOs do not require additional acid treatment or oxidation treatment because a large number of oxygen-containing functional groups have been introduced to GOs in the preparation process.^18,53^ Wang's group first reported the surface modification of CNTs with a plasma technique, and their application in the removal of metal ions.^54,55^ In 2011, the earliest results of GOs for the removal of heavy metal ions (*i.e.*, Co(ii), Cd(ii))^18^ suggested that GOs are very suitable materials for the elimination of pollutants in pollution cleanup. The high dispersion properties of GOs in solutions can greatly increase the interactions of GOs with metal ions, thereby increasing the sorption ability of metal ions to GOs. The oxygen-containing functional groups could avoid the aggregation of GOs and improved the available sorption sites for the binding of metal ions, which increased the sorption capacity of metal ions.^56^ In addition, GOs can be functionalized with metal oxides and organic chemicals, which also could improve the sorption capacity.^47^ The surface electrokinetic potential was dependent on pH values, and also changes the surface properties of carbon nanomaterials and thereby affects the sorption of metal ions.^23^ The kinetic sorption of Cr(iii) on CNTs increased quickly at the initial contact time (<10 minutes), then the sorption equilibration was reached (contact time from 10 to 60 minutes), and at last part of surface adsorbed Cr(iii) was desorbed from CNT surfaces.

**Fig. 11:**
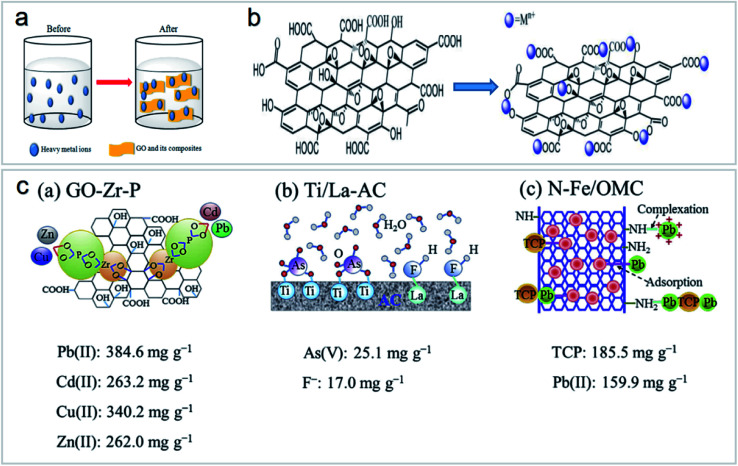
(a) Mechanism of heavy metal ion sorption by GOs and their composites “reproduced from ref. 46 with permission from Elsevier, copyright 2017”, (b) structure of GOs and their interaction with heavy metal ions “reproduced from ref. 46 with permission from Elsevier, copyright 2017”, (c) schematic of functionalized nanomaterials for combined pollutant removal “reproduced from ref. 43 with permission from Elsevier, copyright 2018”.

Heavy metal ion sorption on GOs is mainly dominated by ion exchange, electrostatic attraction, and surface complexation^46,56,57^ (Fig. 11a and b). However, in the real polluted environment, multiple pollutants always coexist. The simultaneous removal of two or more metal ions from wastewater by functionalized carbon nanomaterials is most important for wastewater treatment (Fig. 11c). Most research studies on the application of carbon nanomaterials for the elimination of metal ions are still at the laboratory level. There is still a lack of research on the removal of combined pollutants by carbon nanomaterials, but these studies are very important.^43^

**Fig. 12:**
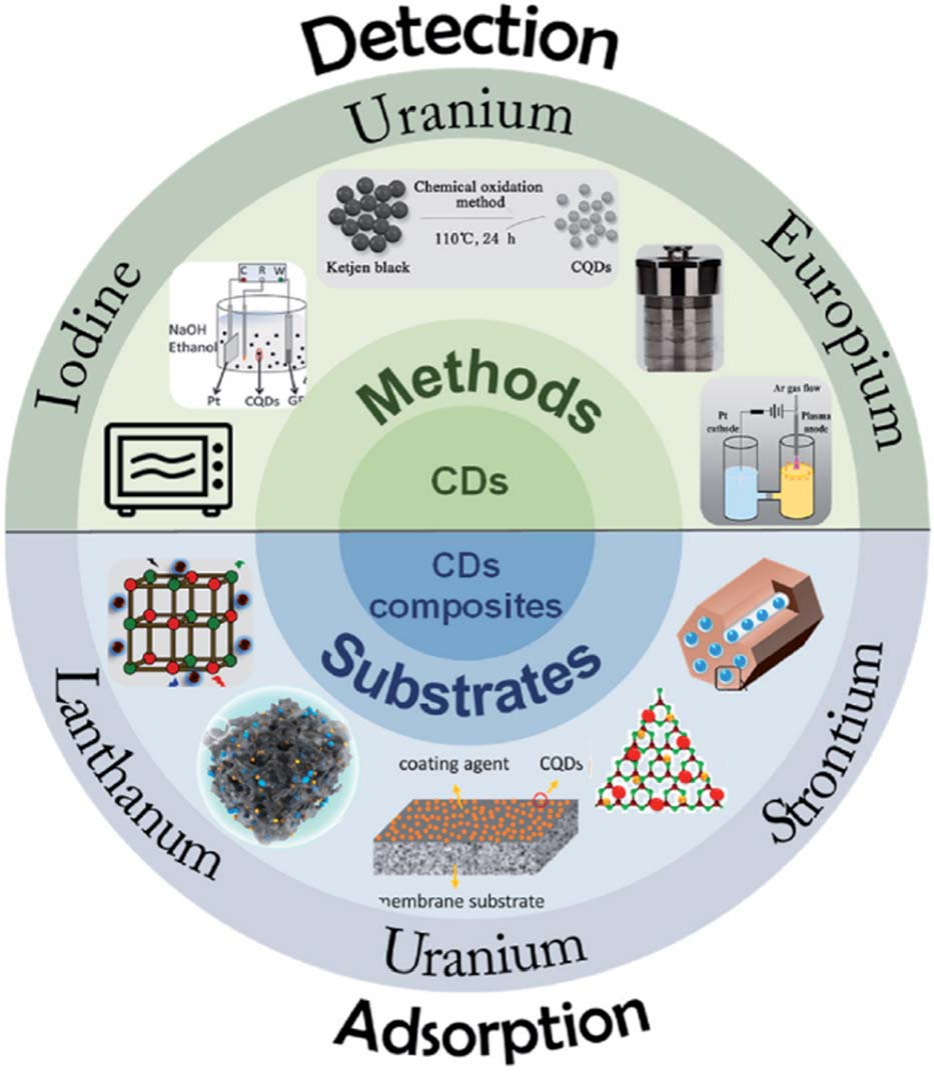
Illustration of CDs and their composite materials for the detection and adsorption of radioactive ions “reproduced from ref. 22 with permission from Elsevier, copyright 2022”.

The application of CDs in the adsorption of U(vi), Eu(iii), Sr(ii) and other radionuclides is reviewed, and the contents indicated that CDs could efficiently remove radionuclides from solutions.^22^ Wang *et al.*^58^ prepared CDs by a microplasma-assisted *in situ* method and introduced them into functionalized ordered mesoporous silica (SBA-NH_2_). The composite retained the high specific surface area of mesoporous silica and the fluorescence properties of CDs. It has high adsorption efficiency for uranium and the adsorption process can be monitored simultaneously (Fig. 12).

Based on the abovementioned results, one can see that carbon-based nanomaterials have high sorption capacity, which is most important for their application in environmental pollution cleanup. With the development of technology, the price for the synthesis of carbon-based nanomaterials on a large scale will decrease. Although the purity of carbon-based nanomaterials is not high enough when they are prepared on a large scale, the high sorption ability ensures their application in the preconcentration of heavy metal ions from large volumes of wastewater. The high surface areas, abundant functional groups and available free sites for the binding of metal ions are most important for the preconcentration of metal ions from aqueous solutions. However, for the highly selective removal of target metal ions from complicated solutions, the surface grafting of special functional groups, which could form selective surface complexes with target metal ions, could improve the selectivity of metal ions binding on carbon-based materials. Another method is the synthesis of nanomaterials with special structures and functional groups, which could selectively adsorb target metal ions.

### 3.2 Adsorption of organic pollutants

Different types of carbon-based nanomaterials have different surface functional groups, which indicate that the interaction mechanism of organic pollutants with carbon-based nanomaterials is relatively different. However, physical adsorption, electrostatic action, π-π action, Fenton/Fenton-like processes and photocatalytic degradation are generally considered the main reactions for the elimination of organic pollutants.^5^ Zhao *et al.*^59^ first reported the application of GOs for the removal of persistent aromatic pollutants (*i.e.*, naphthalene and 1-naphthol) and the results showed an adsorption capacity of 2.3–2.4 mmol g^−1^ for 1-naphthol and naphthalene, which was the highest adsorption capacity of today's nanomaterials, suggesting the great potential of GOs in environmental pollution cleanup. Jeong *et al.*^60^ applied carbon-based nanomaterials including single-walled CNTs (SWCNTs), multi-walled CNTs (MWCNTs) and expanded graphite (EG) in wastewater treatment, and the results showed that MWCNTs had much higher adsorption capacity than EG. The EEM images of Suwannee River Natural Organic Matter (SRNOM) have the typical fulvic acid-like and humic acid-like peaks, at Ex/Em of 220–250/400–450 and 300–350/400–450, respectively. The EEM images of residual SRNOM do not show a notable peak shift. However, the fluorescence intensity decreased. The decrease was more significant for the humic acid-like peak than the fulvic-like peak. Humic acid-like, fulvic acid-like, and SMP-like (Ex/Em 250–300/330–380 nm) peaks were found in EEM images. After the adsorption of SRNOM, the peak intensity of SWCNT greatly decreased. The SMP-like peak disappeared. For MWCNTs, the fulvic acid-like peak decreased more significantly than other peaks similar to the SRNOM results. For EG, the SMP-like peak was slightly decreased, while a uniform decrease of the three peaks was observed. The fluorescence intensity spectra were in good agreement of the EEM images.^60^

Chen *et al.*^50^ studied three kinds of carbon nanomaterials including SWCNTs, oxidized SWCNTs and non-porous graphite powder for the removal of six compounds (thiophene, pyrimidine, benzene, aniline, 2-aminopyrimidine and 4,6-diaminopyrimidine) from aqueous solutions. They found that the affinity of different organic molecules on the three adsorbents varied greatly. The heterocyclic compounds contained N and S sites, which was helpful to improve the adsorption through non-hydrophobic action. The oxidation degree of the adsorbent and the solution pH affected the adsorption of N-containing heterocyclic compounds to the solid phases. The adsorption of organic molecules on carbon-based nanomaterials depends on the properties of organic molecules and the surface properties of nanomaterials. It is well known that the surface properties of materials and the organic molecule properties are dependent on solution conditions, such as pH, ionic strength, temperature *etc.* The properties of nanomaterials are related to the structures, surface functional groups, the synthesis conditions and the precursor used for the preparation of nanomaterials. The competition among different organic molecules in complicated solutions also affects the binding of target organic molecules on nanomaterials.

For the efficient elimination of target organic molecules from wastewater, it is necessary to consider all the above-mentioned parameters to evaluate the suitable materials and best conditions for the selectively and highly efficient elimination of organic pollutants from solutions. The surface properties of carbon nanomaterials are dependent on solution conditions. The properties of organic molecules are also affected by solution conditions. For the application of carbon-based nanomaterials in the elimination of organic pollutants, solution conditions, material properties such as structures and functional groups and organic molecule properties should be taken into consideration.

## 4. Photocatalytic removal of pollutants

Photocatalytic degradation of organic pollutants and sorption/photocatalytic reduction of metal ions under visible light irradiation are useful methods for the efficient degradation of organic molecules or *in situ* solidification of metal ions, especially at low concentrations. In this section, we mainly discussed the photocatalytic degradation of organic pollutants and photocatalytic degradation of heavy metal ions.^60,61^

### 4.1 Photocatalytic degradation of organic pollutants

Although adsorption has been widely used in the treatment of organic pollutants, it also has its disadvantages, such as the re-release of pollutants after the aging of materials. Photocatalytic degradation provides a more efficient solution. Organic molecules can be degraded to carbon dioxide and water under the action of a photocatalyst. In photocatalysis, the absorption of UV/visible light to generate e^−^–h^+^ pairs, the excited charge separation, the transfer of holes and electrons to the catalyst surface, and the generation of active species are necessary for organic molecule degradation. For the effective photocatalytic degradation of organic pollutants, the sorption of organic pollutants on the catalyst is crucial for the degradation reaction of organic molecules by the active species. Carbon-based nanomaterials themselves can be used as photocatalysts. For example, carbon points can degrade rhodamine B (RhB) directly, and benzyl alcohol can be selectively converted to benzaldehyde. After being modified by ZnO, TiO_2_, SiO_2_, CdS, AgNPs, ammonia and sulfuryl chloride, carbon-based nanomaterials can degrade dyes in printing and dyeing wastewater.^62^

**Fig. 13:**
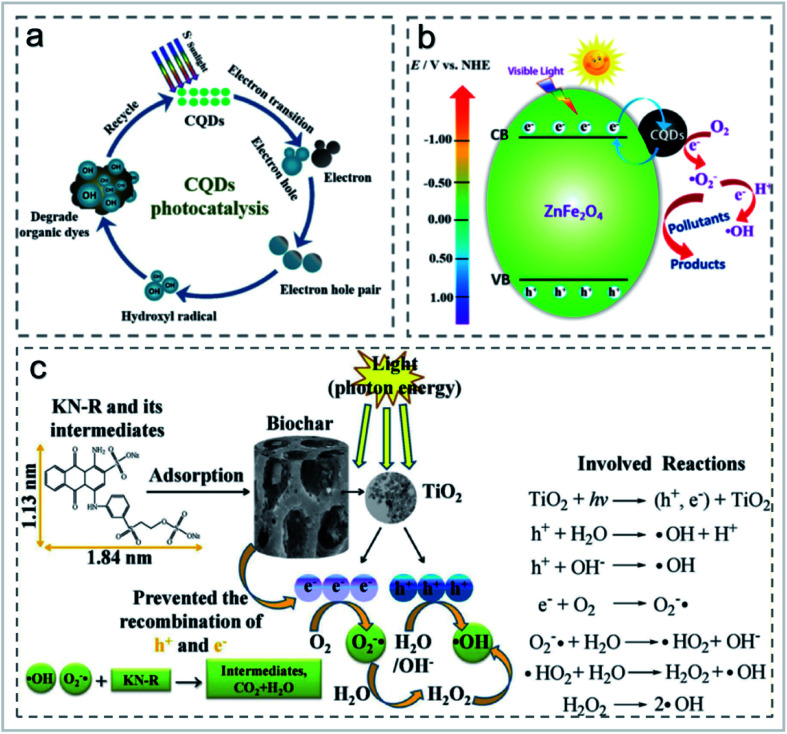
(a) Schematic diagram of the photocatalytic degradation of organic dyes using CDs as photocatalysts “reproduced from ref. 63 with permission from Elsevier, copyright 2019”. (b) The possible reaction mechanism of the CDs/ZnFe_2_O_4_ photocatalyst “reproduced from ref. 66 with permission from American Chemical Society, copyright 2017”. (c) Proposed interaction mechanisms for the decolorization of KN-R “reproduced from ref. 73 with permission from Elsevier, copyright 2018”.

When used for pollutant degradation, CDs were mainly used for photocatalytic degradation of organic pollutants.^63^ CNTs were attached to the surface of porous carbon sponge in a carpet-like vertical arrangement, and used to adsorb methylene blue in wastewater. It was found that the number of CNTs on the sponge determined the adsorption performance of the dyes.^64^ The effect of xenon lamp irradiation on RhB was studied, and it was found that the removal rate of CDs to RhB reaches almost 100% within 120 min under xenon lamp irradiation, and the photocatalytic efficiency gradually improved with the increase of CD content. In the study carried out by Cheng *et al.*,^63^ high quantum yield fluorescent carbon quantum dots (CQDs) were prepared by a hydrothermal method using aqueous mesophase pitch (AMP) as the carbon source. The CQDs were modified by ammonia and thionyl chloride, respectively. The CQDs showed good photocatalytic degradation performance of RhB, methyl blue (MB) and indigo carmine (IC) (Fig. 13a). The degradation rate of N-CQDs to RhB reached 97% in 4 h under natural light irradiation with the degradation rate constant of 0.02463 min^−1^. After repeated use for 5 times, the degradation rate still remained 93%.^63^ Li *et al.*^65^ prepared up-conversion photoluminescence CDs by an alkali-assisted electrochemical method and used it as an efficient near-infrared photocatalyst for selective oxidation of benzyl alcohol to benzaldehyde with a high conversion rate (92%) and high selectivity (100%). Huang *et al.*^66^ prepared a CDs/ZnFe_2_O_4_ photocatalyst by a simple hydrothermal method, whose transient photocurrent response was about 8 times higher than that of the original photocatalyst. Under visible light irradiation, CDs/ZnFe_2_O_4_ nanoparticles can selectively remove gaseous NO_*x*_ and form nitrate (Fig. 13b). The introduction of CDs into metal-free photocatalysts could greatly improve their catalytic performance. Recently, Liu's group prepared a metal-free CDs/g-C_3_N_4_ photocatalyst for the degradation of diclofenac.^67^ The modification of CDs did not have an obvious effect on the morphology and structure of g-C_3_N_4_, while it drastically influenced the optical properties so that the CDs/g-C_3_N_4_ composite could be applied to the photocatalytic degradation of diclofenac.^26^

In the treatment of printing or dyeing wastewater, photocatalytic degradation of dyes is an effective economical technique for the elimination of dyes, especially at low concentrations.^68^ Different kinds of catalysts such as TiO_2_, ZnO, CdS and Ag nanoparticles have been extensively investigated for the photocatalytic degradation of dyes.^69–72^ Zhang and Lu^73^ treated wastewater containing reactive brilliant blue KN-R using a TiO_2_/BC photocatalyst. The decolorization efficiency (81.09%) of KN-R under a UV Xe lamp (300 W) was higher than that of KN-R by a TiO_2_/BC composite without a xenon lamp. Under the condition of no light source, the decolorization rate of active brilliant blue KN-R by original BC reached 65.48%. The original BC showed excellent performance in the decolorization of reactive brilliant blue KN-R, which was due to the appearance of neat large pores with a diameter of 15–20 μm during the adsorption process. When TiO_2_ absorbs enough photon energy equal to or higher than the energy band (*E*_gap_ of TiO_2_ is 3.2 eV), TiO_2_ NPs can generate H^+^ holes and e-electrons. The photogenic hole H^+^ reacted with H_2_O and OH^−^ to generate a hydroxyl radical, and the photogenic electron is captured by O_2_ to generate a superoxide radical (Fig. 13c). The decolorization rate reached 99.7% and 97.0% within 60 min, respectively, under strong acid and strong base conditions. In addition to TiO_2_, Khataee *et al.*^74^ prepared a novel cerium oxide nanoparticle doped BC (CeO_2_–H@BC) composite and applied it for the degradation of active red 84 (RR84) with the degradation rate of 98.5%. The synergistic effect between cerium oxide nanoparticles and BC improved the photocatalytic degradation capacity.^5^ The carbon-based photocatalysts are stable enough and could be applied for the photocatalytic degradation of organic pollutants under visible light conditions, which is critical for the application of carbon-based catalysts in the photocatalytic degradation of dyes.

For organic pollutants at low concentrations, photocatalytic degradation of organic molecules to form the final products (theoretically the best results are CO_2_ and H_2_O) is the best method. The absorption of visible light is most important to improve the photocatalytic ability of the catalyst under visible irradiation conditions. The doping of elements and change of catalyst structure with other materials are good methods to change the catalyst properties such the band gap, and band energy level. The relative contribution of reactive species, the intermediate products in the photocatalytic process, the fast carrier recombination and charge separation/transfer are still unclear now. The stability and reusability of catalysts are the fundamental properties for the application of carbon-based nanomaterials in real applications to avoid possible pollution by the catalysts.

### 4.2 Photocatalytic reduction of metal ions

**Fig. 14:**
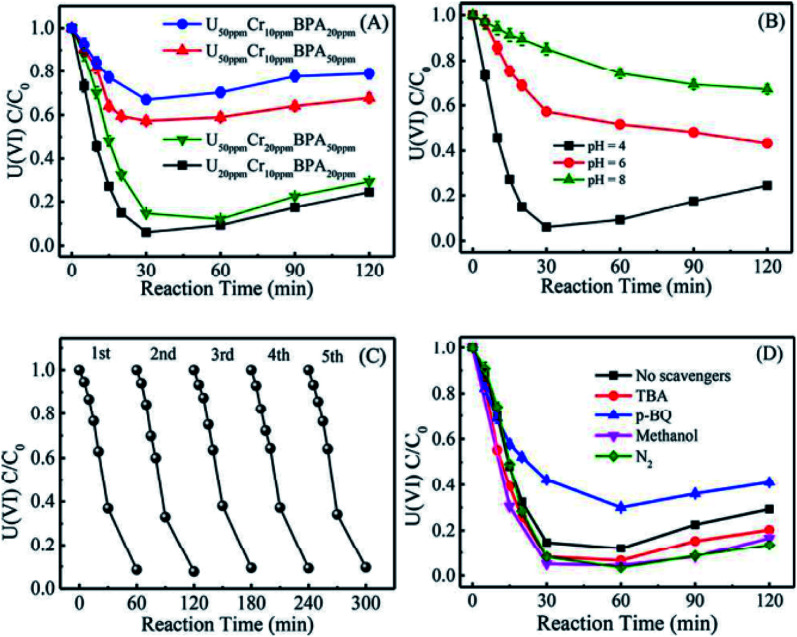
Effect of Cr(vi) and bisphenol A on U(vi) photocatalytic reduction by C_3_N_4_ (A); effect of pH on U(vi) photocatalytic reduction by C_3_N_4_ (B); cycling performance of U(vi) photocatalytic reduction by C_3_N_4_ (C); and effect of different free-radical scavengers on U(vi) photocatalytic reduction by C_3_N_4_ (D) “reproduced from ref. 80 with permission from American Chemical Society, copyright 2019”.

**Fig. 15:**
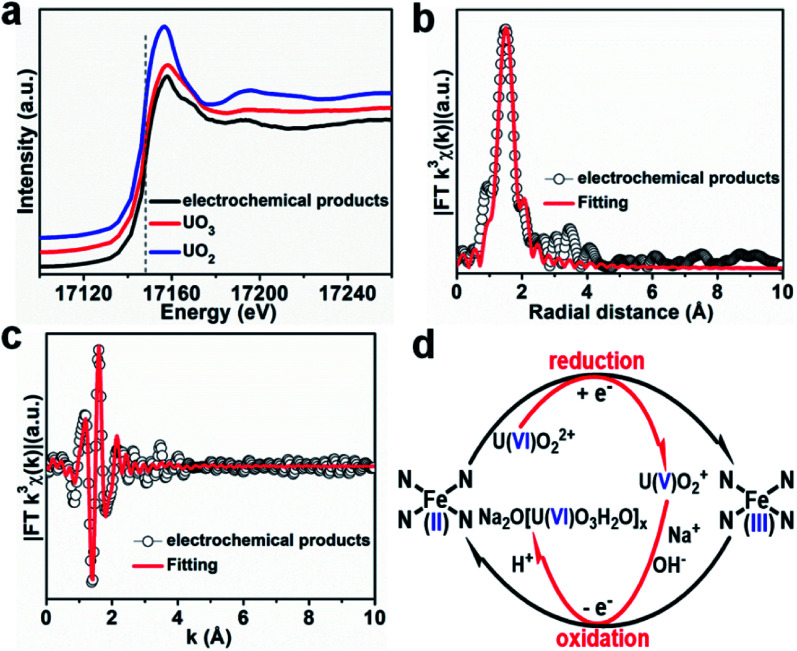
(a) U L_III_-edge XANES spectra of products using Fe–N_*x*_–C–R as the catalyst. (b and c) FT-EXAFS spectra and corresponding *R*-space and *k*-space fitting curves of the products. (d) Plausible reaction mechanism for the Fe–N_*x*_–C–R catalyzed extraction of U(vi) from seawater “reproduced from ref. 82 with permission from Wiley, copyright 2021”.

It is well known that the sorption method is the most efficient approach to remove heavy metal ions from wastewater. However, the sorption technique is not useful in some cases, such as for soil, river, lake, and mine pollution. The solidification and immobilization of metal ions is suitable to decrease the heavy metal ions' pollution. Photocatalytic reduction of a high valent element to a low valent element under visible light irradiation is one of the best methods for the precipitation and immobilization of high valent metal ions such as Cr(vi) to Cr(iii), and U(vi) to U(iv).^75–77^ The sorption-reduction of As(v) by biochar-nZVI showed that As(v) could be reduced by nZVI and then formed precipitates on biochar-nZVI.^78^ The sorption-reduction of Cr(vi) in soil by carboxymethyl cellulose-supported nanoscale iron sulfide biochar composites was studied and the results suggested that Cr(vi) was reduced to Cr(III) to form Cr_2_O_3_ or Cr(OH)_3_. The composites could efficiently immobilize Cr(vi) and converted the more toxic Cr(vi) to less toxic Cr(iii), thereby reducing the bioavailability of toxic Cr(vi).^79^ Wang *et al.*^80^ synthesized C_3_N_4_ and applied it for the sorption-photocatalytic reduction of Cr(vi) and U(vi) in the presence of bisphenol A. The results showed that the presence of bisphenol A and Cr(vi) could enhance the photocatalytic reduction of U(vi) to U(iv). The electron spin resonance analysis and radical scavenger effects showed that the conduction band edge and photogenerated electrons mainly dominated U(vi) photocatalytic reduction, whereas H_2_O_2_ contributed to Cr(vi) photocatalytic reduction (Fig. 14). Li *et al.*^81^ found a new eco-friendly method for the selective and rapid photocatalytic reduction–precipitation of U(vi) without a catalyst, which is useful for the extraction of U(vi) from complicated solutions. In the presence of alcohols and under visible light irradiation, U(vi) could be photocatalytically reduced to U(v) and then transformed into U(iv) through a disproportionation reaction. This finding provided an excellent selective extraction of U(vi) in the presence of other kinds of metal ions and lanthanides. Yang *et al.*^82^ synthesized an iron–nitrogen–carbon catalyst and applied it for efficient extraction of U(vi) from seawater. The amidomine functional groups are specific binding sites for the capture of U(vi), and then the surface adsorbed U(vi) was reduced to U(v) by the FeN_*x*_ centres. The unstable U(v) was oxidized to form solid Na_2_O(UO_3_·H_2_O)_*x*_, which can easily be collected from seawater (Fig. 15).

The sorption-(photo)catalytic reduction–solidification of high valent metal ions to low valent metal ions is an efficient method for the extraction or *in situ* precipitation of high valent metal ions. In the natural environment, it is not necessary to extract metal ions from sediments of rivers or lakes, polluted soils or mines because it is nearly impossible to separate the nanomaterials from the systems. The *in situ* immobilization/solidification of heavy metal ions is one of the best methods to decrease the mobilization of active free metal ions in the environment, and thereby decreases the possible transfer of toxicity of metal ions from the environment to plants or other biological forms. In future, the *in situ* reduction–precipitation of active high valent metal ions is expected to be one of the best techniques to decrease the toxicity of metal ions.

## 5. Interaction mechanism discussion

An understanding of the interaction mechanism of metal ions and organic molecules with carbon-based nanomaterials is helpful to evaluate the potential behavior of pollutants in the environment and also useful for the synthesis of nanomaterials. The sorption mechanism of pollutants from batch techniques is generally evaluated considering the effect of pH and ionic strength. The pH-dependent sorption is mainly dominated by surface complexation, whereas ionic strength-dependent sorption is mainly dominated by ion exchange. At the molecule level, time resolved laser fluorescence spectroscopy analysis could provide the species of metal ions such as the coordination number and H_2_O number in the first coordination sphere, which is helpful to evaluate the formation of inner-sphere or outer-sphere surface complexes. XAFS spectroscopy could provide the unique structure and composition information of metal ions on nanomaterials such as the bond distance, and coordination number, which are helpful to discern the sorption mechanism such as inner-sphere or outer-sphere surface complexation, (co)precipitation, oxidation/reduction *etc.*^83,84^ XPS analysis could provide some information about the interaction of pollutants with functional groups. From the red/blue shift of peak position or appearance of new peaks, one can draw a conclusion about the complexation of special functional groups with pollutant molecules.^85^ Advanced spectroscopy analysis is useful to provide some information about the interaction of pollutants with nanomaterials at the molecule level. More importantly, the combination of several spectroscopy analyses is more important to understand the interaction mechanism.

Besides the advanced spectroscopy analysis, density functional theory (DFT) is an indispensable method to simulate the whole reaction processes. For some reactions, it is difficult/impossible to measure the intermediate products or the contribution of active species from experimental analysis. The DFT calculation could simulate the intermediates, transition states, structures and species of the organic pollutant degradation processes. The interaction energies, bond distances, cluster sizes and radial distribution functions in the possible pathway could also be calculated to understand the possible mechanism. From the results of the batch technique, advanced spectroscopy analysis and DFT calculations, one can draw a conclusion regarding the pollutant molecule interaction mechanism with carbon-based nanomaterials.

## 6. Conclusion and perspective

With a deep understanding of the environmental remediation of pollutants using carbon-based nanomaterials, scientists are paying more and more attention to this field. The synthesis methods and removal mechanism of carbon-based nanomaterials are reviewed herein. According to the material dimension, carbon-based nanomaterials can be roughly divided into four types: zero dimensional materials, one dimensional materials, two dimensional materials and three dimensional materials.

The application of carbon-based nanomaterials in environmental pollution management is mainly realized through adsorption of porous structures and functional groups. For organic pollutants, photocatalytic degradation is an efficient technique for elimination of organic molecules, especially at low concentrations. The final products of CO_2_ and H_2_O are the best results in the photocatalytic degradation of organic molecules. For heavy metal ions, besides the adsorption of metal ions on carbon-based nanomaterials, the photocatalytic reduction of high valent elements to low valent elements, and then the formation of *in situ* precipitates could immobilize the metal ions in the natural environment.

Carbon-based nanomaterials still face the problems of high production cost and difficulty in mass production, leading to the development of most nanomaterials still being in the laboratory stage, which is difficult for large-scale production and use. Nowadays, more and more attention has been paid to environmental pollution control in order to protect the human homeland. It can be predicted that more attention should be paid to cost reduction and mass production in the future research and development of carbon-based nanomaterials. With the development of techniques, carbon-based nanomaterials could be produced on a large scale at a low price. The toxicity of carbon-based nanomaterials in the natural environment should also be considered because they are inevitably released into the environment.

When carbon-based nanomaterials are used in environmental pollution cleanup, it is inevitable that some carbon-based nanomaterials are released into the environment during their synthesis processes and nanomaterial residue in the environment should also be considered. The toxicity of carbon-based nanomaterials should be considered although the research of nanomaterial toxicity in the environment is still at the preliminary stage.

The photocatalytic degradation of organic molecules is still a “black-box” process, especially the analysis of reactive species contribution, and the measurement of intermediate products, which are helpful to evaluate the catalytic properties of nanomaterials. The on-line measurements of intermediates are still very difficult quantitatively measured. Quick on-line analysis may be developed in future to understand the photocatalytic degradation mechanism of organic pollutants.

## Author contributions

Zhixin Liu: investigation, and writing the original draft; Qian Ling: investigation and review; Yawen Cai: writing and review; Linfeng Xu: investigation; Jiahao Su: investigation; Kuai Yu: investigation; Xinyi Wu: investigation; Jiayi Xu: investigation; Baowei Hu: review and editing; Xiangke Wang: writing, review and editing.

## Conflicts of interest

The authors declare that there is no conflict interesting.

## Supplementary Material
